# Determination of Benzocaine in Pharmaceutical Formulations by Indirect SERRS Assay Combined with Azo Coupling

**DOI:** 10.3390/molecules27144492

**Published:** 2022-07-14

**Authors:** Chao-Yang Zhao, Huimin Sui, Endi Xue, Li Li, Jie Zhang, Tao Xu, Xin Liang, Ying Yang

**Affiliations:** School of Pharmacy, Qiqihar Medical University, Qiqihar 161000, China; Zhaocy@qmu.edu.cn (C.-Y.Z.); xed894171756@163.com (E.X.); lilianlinsuo@163.com (L.L.); guanhong@qmu.edu.cn (J.Z.); harvey-333@163.com (T.X.); liangxin@qmu.edu.cn (X.L.); yangying2012@126.com (Y.Y.)

**Keywords:** benzocaine, SERRS, azo coupling, derivatization, Raman

## Abstract

Coupled with an azo coupling reaction, a simple, rapid, sensitive, and effective surface-enhanced resonance Raman scattering (SERRS) detection method for benzocaine was developed. In our study, benzocaine which is used clinically as a local anesthetic was derived with *p*-aminothiophenol into a corresponding azo product within 5 min, resulting in a strong SERRS response with the simple addition of Ag NPs excited with a 532 nm laser. The linear correlation between SERRS intensity of dominant bands and logarithm of benzocaine concentration was investigated for quantitative determination. The method reached a limit of detection (*LOD*) down to 0.139 and 0.0788 μg/mL calculated with two peak intensity ratios (*I*_1568_/*I*_2260_ and *I*_1331_/*I*_2260_), which is comparable to most studies reported previously, and meanwhile had superiority in simplicity and rapidness. The quantitative measurements for pharmaceutical preparations with benzocaine were conducted without complex extraction and enrichment processes. It was indicated that the SERRS assay combined with azo derivatization reaction has implications for practical applications in more complicated systems involving biological samples, in which appropriate and simplified pretreatments were conducted to remove interfering components.

## 1. Introduction

Benzocaine (ethyl 4-aminobenzoate), a local anesthetic, has a prevailing clinical application for local and temporal pain relief of wounds, ulcer surfaces, burns, skin scratches, and hemorrhoids [1]. In 2011, two Food and Drug Administration (FDA) Drug Safety Communications were issued warning that the use of benzocaine, the main ingredients in over-the-counter (OTC) gels and liquids applied to the gums or mouth to reduce pain, can cause methemoglobinemia—a rare but serious condition in which the amount of oxygen carried through the blood stream is greatly reduced related to topical use during medical procedures [2]. The FDA had previously received more than 319 cases of serious adverse events associated with methemoglobinemia after the use of OTC benzocaine products as of April 2011 [3]. Consequently, steps had been taken that OTC oral drug products containing benzocaine should only be used in adults and children 2 years and older, and the companies should add new warning labels for all benzocaine oral health products to describe certain serious risks by the FDA.

Benzocaine is sold in various formulations such as gels, sprays, pastes, ointments, solutions, and lozenges. Moreover, 20% is the maximum content approved in sprays with benzocaine. Despite this, researchers still found that the use of benzocaine spray in endoscopy, intubation, bronchoscopy, and similar invasive procedures may cause methemoglobinemia [4,5,6]. Besides, benzocaine has been used effectively as a general anesthetic for short-term sedation of fish during transport, spawning, and stocking operations in aquaculture of many fishery countries, such as America, Canada, Japan, etc. The US government sets strict limits on the variety, dosage or residue, and withdrawl time of anesthetics [7]. Thus, strengthening the supervision of benzocaine raw material, pharmaceutical preparations by quality assurance detection, and standardized use in aquaculture by conducting residue studies are of great importance. A simple, quick, and sensitive assay for benzocaine is required.

Analytical methods for benzocaine in pharmaceuticals, cosmetics, and complex body tissues have been reported by high-performance liquid chromatography (HPLC) [8,9,10,11,12], electrochemical methods [13,14,15,16], mass spectrometry [9], chemiluminescence [17,18], colorimetric methods [19], and spectrophotometric methods [20,21,22]. Among the current publications we searched, HPLC is predominate as an effective separation technique. An HPLC-UV determination of benzocaine and N-acetylbenzocaine in rainbow trout with a series of acetonitrile, hexane extract, and solid-phase extraction techniques was described, and a limit of detection (*LOD*) of 6 ng/g was obtained [10]. However, they generally suffer from relatively long sample processing or testing time, except for coupling with an electrochemical detector [13]. Several electrochemical methods in recent years were reported with a high detection sensitivity of 2.45 × 10^−6^~3.02 × 10^−8^ mol/L (comparable to 5.00 ng/mL) [13,14,15,16]. These studies mainly focused on the modification of electrodes, involving capsaicin-modified multiwalled carbon nanotube-based electrodes, *p*-chloranil-modified carbon paste electrodes, TiO_2_-GO-modified carbon paste electrodes, and screen-printed electrodes coupled to batch injection analysis (BIA). The electrochemical methods reported need extensive preparation time for electrodes, yet the sample treatment and testing time was greatly shortened. Additionally, a few articles proposed chemiluminescence, colorimetric methods, and spectrophotometric methods based on the diazotization, oxidation, and condensation reaction of benzocaine. Quite different *LOD*s in terms of these studies with various test systems were obtained after extremely simple sample treatment and convenient instrument operation.

A rapid, sensitive, and selective sensing protocol is desirable for the analytes concerned. It will facilitate the accurate study by reducing time delays associated with fabricating the sensing platform and performing an in situ analysis without laborious separation steps. Surface-enhanced Raman spectroscopy (SERS) is a potential analytical technique that meets many of the above characteristics in that it is non-destructive for samples, convenient to operate, highly sensitive to molecules that are adsorbed on or near SERS-active surfaces, and provides unique vibrational fingerprint information of specific molecules [23,24,25,26]. SERS response of a molecule depends primarily on three aspects: (1) the intensity of the electromagnetic field generated by local surface plasmon resonance (LSPR) of noble metal nanostructures; (2) the proximity of the molecule to SERS substrate’s surfaces; (3) the Raman cross-section, or polarizability of the molecule [27]. Therefore, a large body of work has been carried out to make SERS a generalized analytical technique, including preparing highly enhancing SERS substrates and establishing new methods.

To our knowledge, very few studies on SERS-based direct detection of benzocaine with certain *LOD* have been reported [28,29]. They enhanced the SERS signals of benzocaine from the point of preparing self-assembled nanomaterials with high sensitivity and reproducibility. The *LOD*s were 0.9 × 10^−6^ g/cm^2^ (sample on banknotes) and 4.3 × 10^−7^ mol/L, respectively. High sensitivity was achieved with a complicated and time-consuming process of substrate preparation [28] or adsorption between analytes and substrates (~12 h) [29]. A more simple, sensitive, and feasible SERS approach for benzocaine should still be explored.

Herein, we propose a simple surface-enhanced resonance Raman scattering (SERRS) method for the indirect determination of benzocaine based on an azo reaction with *p*-aminothiophenol (PATP). The intensity of SERS bands associated with corresponding chromophores can be selectively enhanced by 2~6 magnitude when the excitation wavelength falls in the electron absorption band, called SERRS [30]. In our study, benzocaine was derived into an azo compound which produces a resonance effect with a 532 nm excitation wavelength, generating a strong SERRS response for indirect quantitative analysis of benzocaine. (1) Short derivatization time (5 min) and mild reaction conditions (common regents, 0 °C, and magnetic stirring) contribute to the simplicity and rapidness of the method. Additionally, Ag NPs, easily prepared by hydrothermal reaction and commonly used in highly sensitive SERS detection, were employed as the active substrate. Immediate Raman measurements were performed once mixing the benzocaine-derived azo product and Ag NPs. (2) Satisfactory detection sensitivity by linear regression between the intensity of dominant SERRS bands and logarithmic value of benzocaine concentration was obtained due to the high sensitivity of SERRS technology. (3) Besides, abundant vibrational information of the azo product indicates the specificity of the method. (4) Finally, the proposed method was applied to real pharmaceutical formulations without time-consuming extraction and enrichment steps. We demonstrate that our azo derivatization-based SERRS assay provides a simple, rapid, sensitive, and effective way for indirect detection of benzocaine in raw materials and pharmaceutical formulations. Furthermore, the approach established here may also be extended to food and cosmetics safety applications such as benzocaine excessive residues in aquatic products and illegal addition in cosmetics.

## 2. Results and Discussion

### 2.1. Characterization of Ag NPs

Ag and Au NPs are the most commonly used SERS-active substrates [31], not only for SERS mechanism studies but also for application research. This is attributed to their physical and chemical advantageous properties, such as the LSPR effect, quantum size effect, catalytic properties, and so on. Generally, Ag NPs exhibit a better SERS response than Au NPs under common excitation sources. Therefore, for the LSPR effect with 532 nm excitation, we used Ag NPs as enhancing substrates to obtain SERRS signals of the benzocaine-derived azo product. The prepared Ag NPs with a diameter of about 47.3 ± 13.0 nm were uniform and monodispersed with a grey-green color. An absorption at 440 nm of Ag NPs was observed (Figure S1) and its TEM image is shown in Figure S2.

### 2.2. Intrinsic SERS Spectra of Benzocaine

**Figure 1:**
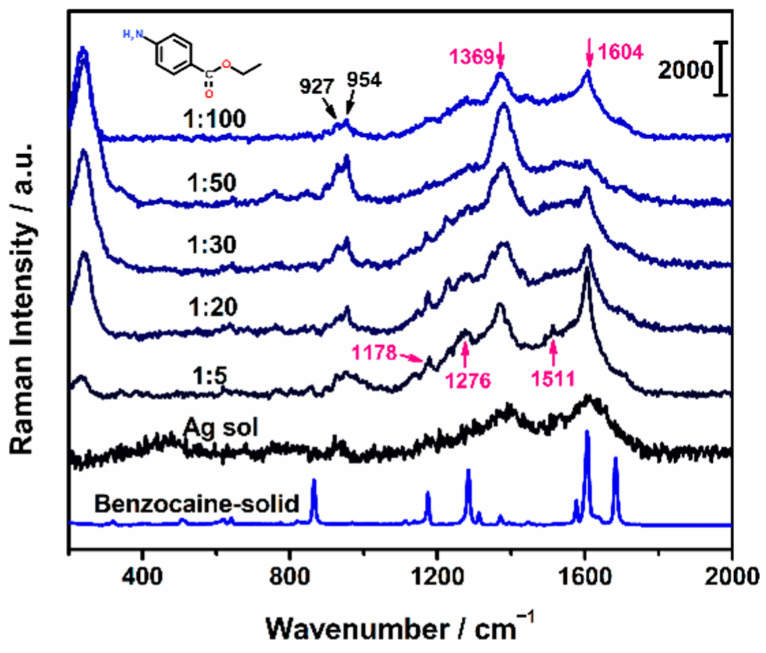
SERS spectra of benzocaine at the concentration of 1 mg/mL with Ag NPs at the volume ratio of 1:100, 1:50, 1:30, 1:20, and 1:5. Raman spectra of benzocaine solid and Ag NPs. Excitation wavelength: 532 nm. The inset is the structure of benzocaine.

Benzocaine belongs to para-aminobenzoic acid esters of local anesthetics. The structure of benzocaine is shown in the inset of Figure 1. Weak SERS signals were obtained when mixing benzocaine stock solution (1 mg/mL) with Ag NPs directly at different ratios. Immediate detection was carried out under the excitation wavelength of 532 nm (Figure 1). The peaks at 1178, 1276, 1369, 1511, and 1604 cm^−1^ were assigned to SERS bands of benzocaine, and several bands (such as 927 and 954 cm^−1^) from the citrate capping agent were also observed [32]. It was demonstrated that performing trace determination for benzocaine by its intrinsic SERS under our experimental conditions was infeasible. Thus, a SERRS assay for sensitive and rapid detection of benzocaine based on an azo coupling reaction was proposed here.

### 2.3. Azo Derivatization of Benzocaine

**Figure 2:**
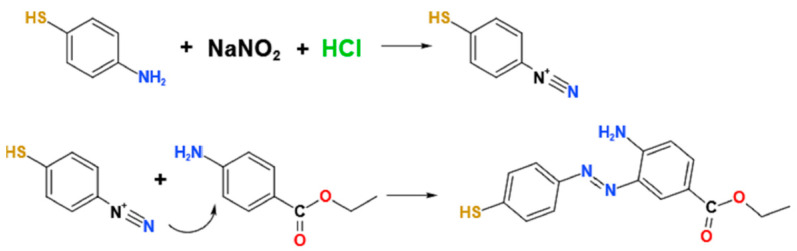
Azo coupling reaction between diazonium ions and benzocaine.

As mentioned above, diazonium ions (-N^+^≡N) were obtained through the reaction between the amino group of PATP and nitrous acid under acidic and low-temperature conditions. According to the mechanism of the coupling reaction, as an electrophilic agent, the diazonium ions can directly attack the ortho position of the phenyl amino group via an electrophilic substitution reaction [33,34]. The possible coupling process is shown in Figure 2.

**Figure 3:**
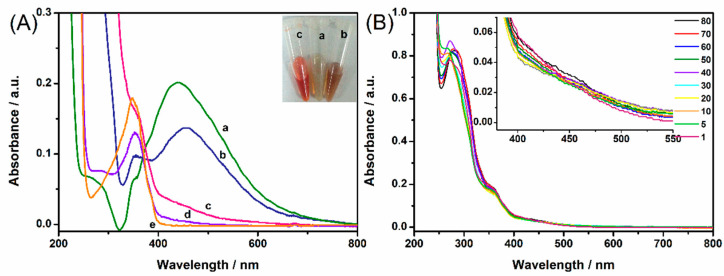
(**A**) UV–vis absorption spectra of Ag NPs (a), Ag NPs-azo mixture at a ratio of 10:1 (b), azo compound derived from 1 mg/mL benzocaine (c), blank sample (d) and diazonium ions from PATP (e). The inset is the visible color change of benzocaine-derived azo product. (**B**) Derivatization time-dependent UV–vis spectra of benzocaine-derived azo solution within 80 min. The concentration of benzocaine was 1 mg/mL and the solutions were diluted at the same multiple.

The solution color was dramatically changed after benzocaine standard solution was added to the diazonium ions solution. The color of the benzocaine-derived azo solution was dark red and clearly visible with naked eyes as shown in the inset of Figure 3A. The color changes were also confirmed by the UV–vis absorption spectrum. The diazonium ions had an absorption peak at 347 nm, and the benzocaine-derived azo solution showed absorption peaks at 400~550 nm. In our study, Ag NPs aggregated to a certain extent when mixed with the produced azo solution with a volume ratio of 10:1 which can be verified by the red shift to 456 nm of the Ag NPs–azo mixture. According to the LSPR effect of Ag NPs and the resonance effect of the azo compound [35], 532 nm was chosen as the excitation laser for the following SERRS measurements. Meanwhile, as mentioned in our previous work, when the diazonium ions were prepared from PATP, the blank solution had similar but much weaker absorption with the benzocaine-derived azo product, which was possibly due to the slight self-coupling reaction of the raw material molecule—PATP [36].

### 2.4. Optimization of Derivatization Time and Stability Investigation of Azo Product

As shown in Figure 3B, derivatization time-dependent UV–vis absorption spectra representation of the benzocaine-derived azo product was conducted. All the absorption values in the range of 400~500 nm were basically unchanged during 80 min. From the above results, 5 min was adopted as the azo derivatization time in our study in order to ensure high reaction efficiency and greatly shorten the detection time for the purpose of rapid detection.

### 2.5. Mixing Ratio Optimization of Azo Solution and Ag NPs

**Figure 4:**
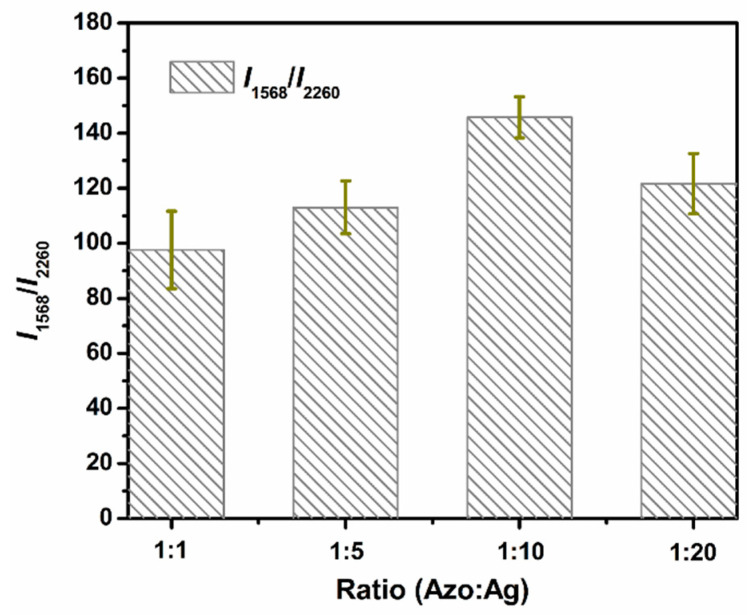
Mixing ratio of azo solution and Ag NPs-dependent SERRS intensity ratio (*I*_1568_/*I*_2260_). The azo solution was derived from 1 mg/mL benzocaine. Excitation wavelength: 532 nm. The error bars were made according to at least three data sets.

The enhancement ability of active substrates to analytes varies with their mixing ratios due to different adsorption states. The optimization experiment on the mixing ratio of the azo solution derived from 1 mg/mL benzocaine stock solution and Ag NPs was carried out (Figure 4). SERRS measurements were performed for the azo–Ag NPs complex with the volume ratios of 1:1, 1:5, 1:10, and 1:20. Several characteristic peaks including 1076, 1387, 1439, and 1568 cm^−1^ were relevant to the azo product. The peak at 2260 cm^−1^ from acetonitrile served as the internal standard. We adopted the SERRS intensity ratio (*I*_1568_/*I*_2260_) to represent the enhancement effect of Ag NPs for the resultant azo product. The error bars were made according to at least three sets of parallel tests. It was observed that the relative intensity of 1568 cm^−1^ to 2260 cm^−1^ increased at first and then decreased with the increase in Ag NPs volume ratio, reaching the highest value at 1:10. Thus, the mixing ratio of the azo product and Ag NPs was optimized to 1:10 in the following experiment.

### 2.6. SERRS Spectra of Azo Product

**Figure 5:**
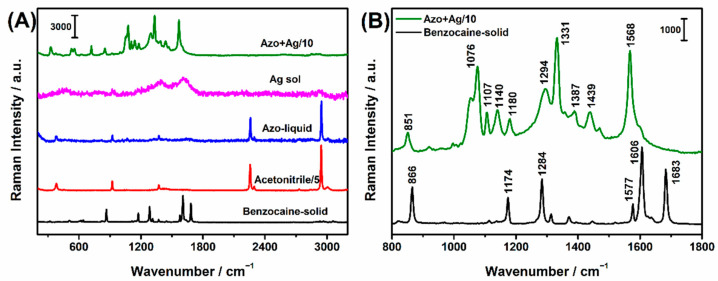
(**A**) Raman spectra of Ag NPs, benzocaine-derived azo solution, acetonitrile, benzocaine solid, and SERRS spectrum of benzocaine-derived azo solution. (**B**) Specific peak positions for Raman spectrum of benzocaine solid and SERRS spectrum of azo compound derived from 1 mg/mL benzocaine with Ag NPs at the volume ratio of 1:10. Excitation wavelength: 532 nm.

We also investigated the Raman responses of some substances involved in our study in Figure 5A. No obvious peaks were observed for Ag NPs; therefore, there would be no interference with subsequent quantitative analysis for benzocaine. When the energy of excitation light is equal to or close to the spacing energy between two electron energy levels in a molecule, the excitation light is strongly coupled with electrons, that is the resonance effect. In addition, the corresponding vibrational bands will be enhanced selectively, producing a resonance Raman effect. In our previous study, a resonance Raman spectrum of an azo compound can be likely characterized [36,37]. However, in this experiment, no characteristic peaks assigned to the azo product appeared probably due to the masking of solvent–acetonitrile or molecular differences. It was indicated that the azo product in low concentrations could be hardly determined only by the resonance Raman technique. As we all know, Ag NPs yield strong Raman enhancement and have been applied in biology, medicine, material, and other fields extensively. Herein, Ag NPs were induced into the detection system as active substrates to produce a strong SERRS response for sensitive detection. Markedly enhanced SERRS signals were observed owing to the resonance effect of the azo product and the LSPR effect of Ag NPs.

Specific peak positions were represented in Figure 5B. For benzocaine solid, the strong bands including 866, 1174, 1284, 1577, 1606, and 1683 cm^−1^ were simply assigned [38]. The band at 866 cm^−1^ was assigned as the bond stretching mode of the phenyl ring, CO stretching coupled with COC in-plain bending modes in the ethyl-ester chain. The band at 1172 cm^−1^ came from in-plain CH bending (ring) and phenyl ring stretching vibration. The peak of 1284 cm^−1^ was attributed to CC and CO stretching vibration of the ethyl-ester chain. The band at 1577 cm^−1^ was from phenyl ring stretching coupled with in-plain CH angel bending modes within the phenyl ring. The very strong band at 1606 cm^−1^ was assigned to ring stretching and in-plain CH bending vibrations coupled with NH_2_ scissoring vibration. Another very strong band observed at 1683 cm^−1^ was assigned as C=O stretching vibration mode from the carbonyl group. For the benzocaine-derived azo compound, massive fingerprint vibration information was collected. The band at 1439 cm^−1^ was assigned as N=N stretching vibration in the trans form [39], indicating the formation of the azo product. The very strong bands included 1076, 1331, and 1568 cm^−1^. Among them, the band at 1331 cm^−1^ was due to CCH bending and NCC bending modes within the phenyl ring. The band observed at 1568 cm^−1^ was assigned to CC stretching vibration within the phenyl ring. Besides, the band at 1387 cm^−1^ was assigned as CC stretching and CH bending modes within the phenyl ring [33,34,39]. According to the selection rules of SERS [40], it was inferred that phenyl groups tend to be perpendicular to the Ag NPs surfaces. Therefore, it should be presumed that the benzocaine-derived azo compound was possibly absorbed onto the Ag NPs surface through both the SH group of PATP and the NH_2_ group of benzocaine.

### 2.7. Concentration Dependent SERRS Characterization and Linear Investigation

A certain degree of aggregation appeared immediately once the azo solution was added to Ag NPs by naked eyes (inset of Figure 3) and by confocal microscopy of the Raman spectrometer due to high concentrations of inorganic salts used in the reaction and the interaction between azo and Ag NPs. Further aggregation occurred under laser irradiation, inducing a stronger LSPR effect and then higher SERS enhancement.

**Figure 6:**
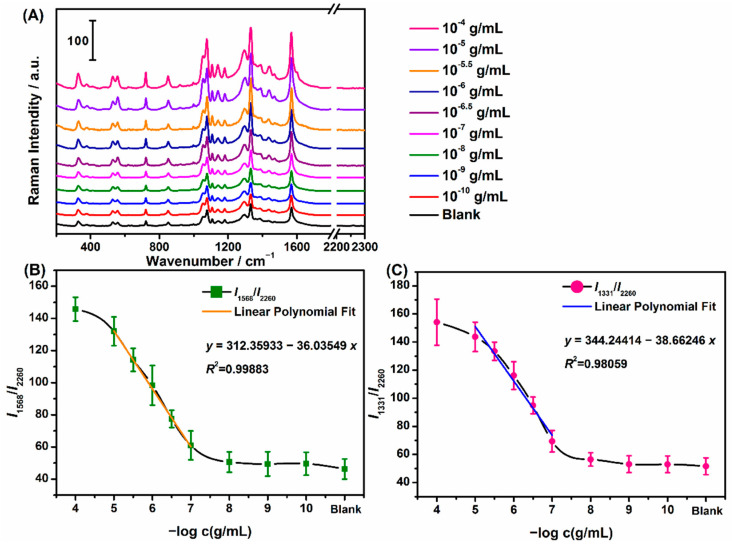
(**A**) Benzocaine concentration-dependent SERRS spectra of the corresponding azo compound. The intensity ratio of SERRS peaks at 1568 cm^−1^ (**B**) and 1331 cm^−1^ (**C**) to 2260 cm^−1^ versus the negative log concentration of benzocaine. Each error bar indicates the standard deviation of the SERRS intensity ratio. The error bars were made with at least five sets of data.

Representative concentration-dependent SERRS spectra of the benzocaine-derived azo–Ag NPs complex and the plot of SERRS intensity ratio (*I*_1568_/*I*_2260_, *I*_1331_/*I*_2260_) versus the negative log value of benzocaine concentration are given in Figure 6. The SERRS spectra were first baselined and then normalized by a peak intensity of 2260 cm^−1^ to minimize systematic errors. As can be seen from Figure 6A, the intensities of bands at 1331, 1387, and 1568 cm^−1^ decreased gradually with the benzocaine concentration coming down. It was noted that there were certain background signals for the blank sample which were basically consistent with the UV–vis absorption results due to the possible self-coupling of PATP. The variation tendencies of SERRS intensity ratios (*I*_1568_/*I*_2260_, *I*_1331_/*I*_2260_) with negative logarithm concentration of benzocaine were investigated in Figure 6B,C. The error bars were made with at least five sets of data which represented the precision. Finally, after linear fitting by Origin 8.5, the results showed that the linear equation was *y* = 312.35933 − 36.03549*x* (*R*^2^ = 0.99883) and *y* = 344.24414 − 38.66246*x* (*R*^2^ = 0.98059) in the range from 0.01 mg/mL to 0.1 μg/mL, respectively. The *LOD*s were calculated to be 0.139 and 0.0788 μg/mL (*S*/*N* = 3). The *LOD*s had reached nanogram levels, in which it turned out that the proposed SERRS assay based on the azo coupling reaction was feasible for quantitative analysis of benzocaine at trace levels with sensitivity, simplicity, and rapidness.

### 2.8. Accuracy and Reproducibility

In order to verify the accuracy and reproducibility of the resulting standard curve, the recovery and repeatability tests were conducted by preparing three samples in different concentrations within the linear range, obtaining corresponding SERRS signals (each concentration was detected five times). Then recovery was calculated by the ratio of measured concentration and true concentration. As shown in Table S1, the recoveries were 95.1~103.4%, and the intra-day and inter-day *RSD*s were within 11%, demonstrating the precision and repetition of the method.

### 2.9. Determination in Real Samples

The practical applicability of the newly developed method quantified by 1568 cm^−1^ was tested on pharmaceuticals with three labels. Five replicate determinations were conducted using the proposed procedure and the results were summarized in Table 1.

### 2.10. Comparison with Other Techniques for Benzocaine

As shown in Table 2, we summarized current detection methods for benzocaine in the literature, including sample treatment procedure, instrument test time, *LOD*, or linear range. HPLC-UV methods generally suffer from relatively long sample processing or testing time, except for coupling with an electrochemical detector [13]. Electrochemical methods could get better *LOD*s [15] than HPLC-UV methods with extensive preparation and modification time for electrodes. For chemiluminescence [17,18], colorimetric methods [19], and spectrophotometric methods [20,21,22], their sample treatment processes were much more simple. In our proposed method, the sample treatment (ultrasonicated 5 min for gel and enema, diluted for spray directly) is simple compared with HPLC methods. Additionally, the azo coupling time is very short (5 min), supporting rapid detection without time-consuming substrate preparation procedures as electrochemical methods. Thus, our method is simple and rapid in sample treatment and instrument operation without a complex preparation time of sensors. Furthermore, the *LOD* of our method-0.139 μg/mL (equates to 8.39 × 10^−7^ mol/L) is comparable to and better than many current methods in the literature.

## 3. Materials and Methods

### 3.1. Chemical Reagents

Benzocaine, PATP, and silver nitrate were purchased from Sigma-Aldrich (Shanghai) Trading Co., Ltd., Shanghai, China Pharmaceuticals, namely Lidinuo compound benzocaine gel (labeled to contain 1 g benzocaine and 5 mg zinc chloride per 5 g), were purchased from a local pharmacy. Enemeez (mini-enema, from the makers of DocuSol, labeled to contain 283 mg of docusate sodium and 20 mg of benzocaine per 5 mL) and Americaine benzocaine topical anesthetic spray (Insight Pharmaceuticals LLC, Langhorne, PA, USA, labeled to contain 20.00% benzocaine, 57 g) were purchased from the Internet. The above reagents were stored in the refrigerator at 4 °C. Sodium carbonate, hydrochloric acid, sodium citrate, and sodium nitrite were obtained from Beijing Chemicals Co., Ltd., Beijing, China. All these chemicals were analytical-grade reagents and were used directly without further purification. The purified water purchased from Wahaha Group Co., Ltd., Hangzhou, China, was used throughout the experiment.

### 3.2. Apparatus and Measurement

Raman spectra were measured with a Thermo Scientific DXR3xi Raman Imaging Microscope equipped with an excitation laser wavelength of 532 nm (laser power of 10.0 mW) at room temperature. All Raman spectra were recorded by a 10× microscope objective. The exposure time was set as 0.5 s with 10 scans. The pinhole was 25 μm. The Raman spectra were obtained through capillary by wicking action and then baseline corrected by NGS LabSpec 5, normalized by peak intensity of 2260 cm^−1^ from acetonitrile with Origin 8.5. UV–vis absorption spectra were recorded with a T6 UV-Vis spectrophotometer (Beijing Purkinje General Instrument Co., Ltd., Beijing, China). Medium scanning speed was used and the scanning interval was set as 0.2 nm. All solutions characterized by the UV–vis spectrophotometer were diluted a certain number of times in order to avoid excessive absorption values. TEM image of Ag NPs was recorded on a JEOL JEM-2100F field transmission electron microscope.

### 3.3. Synthesis of Ag NPs

The Ag NPs were prepared according to the modified Lee and Meisel method [31]. Sodium citrate (2 mL, 1%, *w*/*v*) was rapidly added into silver nitrate solution (2 × 10^−3^ mol/L) in the state of slightly boiling under magnetic stirring and the solution was kept at 85~90 °C for 40 min. The produced silver colloid in grey-green color was naturally cooled to room temperature and then stored in the refrigerator at 4 °C.

### 3.4. Derivatization Method of Benzocaine

Firstly, 10.00 μL sodium nitrite solution (5%, *w*/*v*) was added to 10.00 μL *p*-aminothiophenol solution (1.000 × 10^−3^ mol/L, the solvent was 0.12 mol/L hydrochloric acid) in an ice-water bath with magnetic stirring. One minute later, 20.00 μL benzocaine solution to be tested and 10.00 μL sodium carbonate solution (10%, *w*/*v*) were rapidly added into the above diazonium salt solution for another 5 min to obtain the corresponding azo compound. The comparative experiment was carried out by replacing benzocaine solution with an equal volume of solvent according to the above experimental conditions. The produced azo solution was immediately characterized by the UV–vis spectrophotometer and Raman spectroscopy.

### 3.5. Preparation of Benzocaine Standard Solution

All the benzocaine solutions were prepared with a mixed solvent of acetonitrile and water at a ratio of 16:9. Benzocaine stock solution was prepared by weighing 0.1000 g benzocaine precisely and dissolving in a 100 mL volumetric flask with the above-mixed solvent. Other benzocaine standard solutions were obtained by the stepwise dilution method with the concentrations of 0.1 mg/mL, 0.01 mg/mL, 1 μg/mL, 0.1 μg/mL, 0.01 μg/mL, 1 ng/mL, and 0.1 ng/mL, respectively. All solutions were freshly prepared and stored at 4 °C for further use.

### 3.6. Sample Preparation

Four grams of gel was accurately weighed and dissolved with 50 mL acetonitrile in a 100 mL Erlenmeyer flask and ultrasonicated for 5 min. Appropriate dilution with acetonitrile and water was conducted to make sure the final solvent was in the same proportion as the above-mixed solvent. Four grams of the enema was treated the same way as the gel. Four milliliters of the spray was directly dissolved and diluted without ultrasonic treatment. The final concentration of benzocaine was approximate 5 μg/mL. All the solutions were stored at 4 °C.

## 4. Conclusions

A new, simple, quick, sensitive, and effective SERRS analytical method combined with an azo coupling reaction has been developed to be applied to indirectly determinate benzocaine in gel, enema, and spray. The whole experiment process is convenient by simply mixing different reagents followed by SERS operation. The reaction time for azo coupling is only 5 min which ensures the rapidity of the method. The sensitivity depends on the resonance effect of the azo product under 532 nm excitation wavelength and the LSPR effect induced by Ag NPs. Furthermore, the selectivity is guaranteed by unique SERRS fingerprint vibrational information. In conclusion, the SERRS assay based on azo coupling provides a simple, quick, sensitive, and selective platform for quantity control of benzocaine without expensive and time-consuming enrichment processes. The advantages of the proposed methodology give it the potential to be used for benzocaine determination in more complex systems.

## Figures and Tables

**Table 1:** Content of benzocaine in selected pharmaceuticals, determined using the method in this work together with content declared by the manufacturer.

Pharmaceutical	Benzocaine Content by Our Method (g/g or mg/mL)	Declared Benzocaine Content (g/g or mg/mL)	*RSD* (%)
Lidinuo compound benzocaine gel	0.210 ± 0.013	0.2	6.2
Enemeez	3.98 ± 0.21	4	5.3
Americaine benzocaine topical anesthetic spray	0.201 ± 0.018	0.2	9.0

**Table 2:** Summary of current detection methods for benzocaine in the literature.

Methods	Samples	Sample Treatment	Instrument Test Time	*LOD* or Linear Range	Reference
HPLC-DAD	Bioadhesive gel	Stirred 90 min, ultrasonicated 10 min	30 min	/	[8]
HPLC-UV-DAD HPLC-ESI-MS	Cosmetic creams	Dispersed, ultrasonicated 10 min	30 min	1.8 μg/g	[9]
				1.7 ng/g	
HPLC-UV	Edible fillet tissue from rainbow trout	Inorganic salt treatment, a glass extraction column and a Baker SPE-24G column, rotary evaporation. Then solid-phase extraction	7.5 min	6 ng/g	[10]
RP-HPLC-UV	Suppository	Extracted three times, centrifuged 5 min	5 min	0.22 μg/mL	[11]
HPLC-UV	Cream	Ultrasonicated 10 min, centrifuged 20 min. Repeated three times.	2.5 min	6~15.6 μg/mL	[12]
HPLC-ED FIA-ED (electrochemical detection)	Pastilles and mouthwash	Dissolved then diluted	10 min	2.0 × 10^−7^ mol/L	[13]
			12 min	1.9 × 10^−7^ mol/L	
TiO_2_-GO/CPE-based ED	Ear drops, tablets, and oral fluid	Dissolved and diluted. Oral fluid: centrifuged 5 min	/	2.48 × 10^−7^ mol/L	[14]
BIA-amperometric method	Fish fillets	Freeze-drying, vortexed 5 min and centrifuged 5 min, frozen and thawed	/	3.02 × 10^−8^ mol/L	[15]
Capsaicin-modified MWCNT and *p*-chloranil-modified CPE-based ED	Analytical grade chemicals	Dissolved and diluted	/	2.45 ± 0.2 μmol/L	[16]
Chemiluminescence (MnO_4_^−^,H^+^)	Spray	Dissolved and diluted	/	0.03 μg/mL	[17]
SIA-CL (MnO_4_^−^,H^+^)	Babydent STADA solution	Diluted	120 h^−1^	0.3 μg/mL	[18]
Colorimetric method	Dentocalm ointment	Extract four times, evaporated to dryness, then dissolved. React for 10 min		10~60 μg/mL	[19]
Kinetic-spectrophotometry (Stopped-flow method)	Tablets and solutions	Dissolved and diluted	100 h^−1^	30 ng/mL	[20]
First-derivative spectrophotometric method	Tablets	Dissolved then diluted	/	10~25 μg/mL	[21]
Surfactant-enhanced spectrophotometric method	Throat lozenges and lozenges of benzocaine	Dissolved, diluted, react 20 min	/	0.0825~4.9558 μg/mL	[22]
SAM nanocube-based plasmene nanosheets for SERS	Banknotes	Dissolved	/	0.9 × 10^−6^ g/cm^2^	[28]
Gold film over PS spheres for SERS	Benzocaine in analytical grade	Dissolved	/	4.3 × 10^−7^ mol/L	[29]
Our method	Gel, enema, and spray	Dissolved, ultrasonicated 5 min (not for spray). React 5 min		0.139 μg/mL (8.39 × 10^−7^ mol/L)	

## Data Availability

Not applicable.

## Supplementary Materials

The following supporting information can be downloaded at: https://www.mdpi.com/article/10.3390/molecules27144492/s1, Figure S1: UV–vis absorption spectrum of Ag NPs; Figure S2: TEM image of Ag NPs and histogram of Ag NPs size distribution; Table S1: Recovery and repeatability tests of the standard curve for benzocaine.

## Sample Availability

Samples of the compounds are available from the authors.
