# Supplementary material for: Determination of Benzocaine in Pharmaceutical Formulations by Indirect SERRS Assay Combined with Azo Coupling
Source: Molecules. 2022 Jul 14;27(14):4492. doi: 10.3390/molecules27144492 (PMC9321682; doi:10.3390/molecules27144492)
Supplement: Supplementary file 1 [file molecules-27-04492-s001.zip › molecules-1795593-supplementary.pdf]

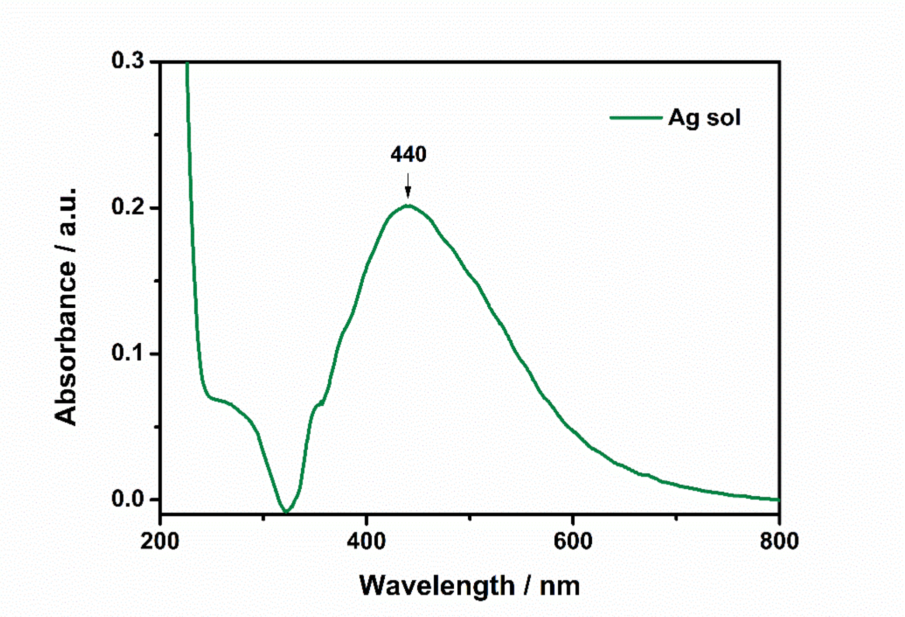

Figure S1. UV-Vis absorption spectrum of Ag NPs.

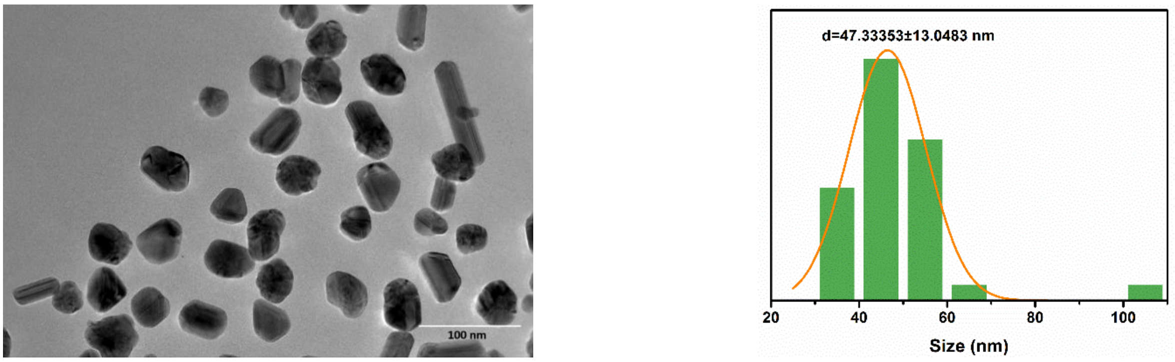

Figure S2. TEM image of Ag NPs (left). The acceleration voltage is 200 kV with the magnifying power of 100000x. Field of view width is 521.1 nm; Histogram of Ag NPs size distribution (right).

Table S1. Recovery and repeatability tests of the standard curve for benzocaine.

True concentration (μg/mL)	Recovery (%)	Intra-day precisions *RSD* (%)	Inter-day precisions *RSD* (%)
0.500	98.5	11	5.3
2.00	103.4	7.4	4.4
7.00	95.1	6.2	5.9

The inter-day precisions were conducted in three days.
